# Unanswered questions in childhood idiopathic nephrotic syndrome

**DOI:** 10.12861/jrip.2014.01

**Published:** 2013-11-02

**Authors:** Ali Lanewala, Muhammed Mubarak

**Affiliations:** ^1^Pediatric Nephrology Department, Sindh Institute of Urology and Transplantation (SIUT), Karachi, Pakistan; ^2^Histopathology Department, Sindh Institute of Urology and Transplantation (SIUT), Karachi, Pakistan

**Keywords:** Nephrotic syndrome, Calcineurin inhibitors, Minimal change dise

**Implication for health policy/practice/research/medical education:**

Recent advances in molecular genetics have helped in unraveling the genetic component of idiopathic nephrotic syndrome in children. Similar advances in understanding the environmental triggers and immune defects will help better understand the pathogenesis of the disease. It is hoped that the above advancements will ultimately lead to targeted therapy and cure of the disease.

The advent of improved technology in genetics has helped to understand better the filtration barrier of kidneys. Newer proteins are being described and defects in genes encoding them are identified. Various mutations in these genes have been associated with a variety of filtration barrier defects (1,2). However, the mechanism of disease still remains poorly understood (3). We know that nephrotic syndrome (NS) is a disease of the immune system because it gets better in a vast majority by different immune suppressive medications (4). Clinically, NS is classified based on either the age of presentation into congenital, infantile, childhood or adolescent type or according to its steroid response into steroid sensitive, dependent or resistant variety (4,5). It is well established through clinical trials that steroid dependent variety responds favorably to cyclophosphamide and steroid resistant to cyclosporine. We are of the view that the key to establish the mechanism of NS is to find out how steroids, cyclophosphamide and cyclosporine modulate the immune system. We know that steroids and calcineurin inhibitors act by inhibiting the signals of lymphocytes to proliferate and communicate. What we do not know is the fact that which specific signals are blocked by cyclosporine that help to treat steroid resistant variety that remain unblocked by steroids. Similarly, cyclophosphamide course brings sustained remission in almost three fourth of children who are otherwise dependent on steroids. The hypothesis of some permeability factor being secreted by the lymphocytes should be focused more intensely. Is it just one substance or is it a group of different chemicals secreted by different subtypes of immune cells?

Another interesting and perplexing clinical observation is that there are different morphological findings in each clinical group (6). Minimal change or focal segmental sclerosis can be steroid sensitive, dependent or resistant. How is it that similar symptom complex and treatment response is seen in different histopathologic lesions? If NS is caused by a permeability factor or factors, then what triggers the different morphological lesions? Whether the deposition of specific or mixed immunoglobulins in the glomeruli is a cause or effect of these different morphological lesions?

We are hopeful that the above questions that seem like a mystery will be answered soon and then perhaps the discovery of the cure of childhood NS will be possible also.

## Authors’ contributions

All authors wrote the manuscript equally.

## Conflict of interests

The authors declared no competing interests.

## Ethical considerations

Ethical issues (including plagiarism, data fabrication, double publication) have been completely observed by the author.

## Funding/Support

None.
